# The Detection of the Bacillus Tuberculosis1Read before the Chicago Medical Society on February 18.

**Published:** 1889-03

**Authors:** Frank Billings

**Affiliations:** 235 State Street


### THE DETECTION OF THE BACILLUS TUBERCULOSIS.[1]

BY FRANK BILLINGS, M.D.

The form of the bacillus tuberculosis is not characteristic and it cannot, therefore, be differentiated from other pathogenic and non-pathogenic bacteria by its form alone. It is a very thin bacillus, about two to five micromillimetres in length (from one-fourth to one-half the diameter of a red blood-corpuscle). It is usually slightly bent.

Like all protoplasmic cells it has an affinity for the aniline colors, and its reaction to these colors is characteristic when the aniline is combined with a mordant.

To elicit the characteristic reaction of the bacillus to the aniline colors it is necessary to proceed in a methodical manner: The instruments used, forceps, needles, etc., should be clean, sterilized by heating in a gas or other flame. The cover glasses and slides should be cleaned in fine alcohol.

The material supposed to contain the bacilli should be collected in a clean vessel, and when collected should be protected from contamination by the air, etc.

The material should be spread in a very thin layer upon a cover glass, by means of a needle or by placing a small amount upon one glass and then pressing another cover glass upon the first, thus making a thin layer upon two cover glasses. The thin film is then allowed to dry upon the cover glass, or the drying may be hastened by warming it over a gas flame. Then, when dry, by passing the cover glass quickly two or three times through the flame, the albumin usually present in the medium fixes the film upon the glass.

The cover glass is now ready for the aniline dye. One may use any color, but aniline violet, methyl blue, or fuchsin is usually employed. Fuchsin is the most often used, because its bright red renders the bacilli more prominent to most observers; and too, one may use with it, better than with the other colors, a contrast color for the ground substance on the cover glass.

The color used must be combined with a mordant, which so fixes it in the bacillus of tuberculosis as to render it very much less susceptible to the bleaching effects of the mineral acids, while it does not so affect other bacteria, with but two exceptions, which I shall mention later.

There are several substances that may be used as a mordant; aniline water, carbolic acid, tannic acid, and others. Aniline water was first used and is still by some, but the mordant now in common use and the one used by Professor Koch, is carbolic acid. It has the advantage over aniline water that a solution of it with the color may be kept indefinitely, while the aniline water solution must be made each time it is used.

The following solution of fuchsin (Ziehl & Neelson) is a satisfactory one in every way:

| | |
|---|---|
| Take of Fuchsin .......... | 1 part |
| Acidi carbolici....... | 5 parts |
| Alcoholis........... | 10 parts |
| Aquæ dostilatæ..... | 100 parts |

Mix in the order given. A few drops of the staining fluid are placed upon the cover glass, held in a forceps with the film upwards over a gas or alcohol lamp, Bunsen's flame, until the solution boils or gives off steam. It is then washed in water and is ready for the process of bleaching.

For bleaching, any of the mineral acids may be used. A twenty-five to thirty-three per centum watery solution of hydrochloric, nitric or sulphuric acid is used. Koch prefers nitric acid; his laboratory assistant uses sulphuric, while at Vienna hydrochloric is chiefly used. It is probably immaterial which acid is employed.

The stained cover glass is immersed in the acid solution for a moment, then in a seventy per centum water solution of alcohol, and finally washed in water. The immersion in acid, alcohol and water suc-

[1] Read before the Chicago Medical Society on February 18.

cessively being repeated until the color is almost or quite bleached. This process leaves the bacillus tuberculosis colored red while the ground substance and all other bacteria, with the two exceptions mentioned, are bleached. The mordant used enables the bacilli of tuberculosis to retain the color. Too long immersion in the acid will also overcome the action of the mordant and render the examination nil. The cover glass should be finally thoroughly washed in water to remove all acid, otherwise the slight amount of acid remaining will gradually fade the color and in a few months the preparation will become worthless.

The cover glass may now be mounted on a slide in water or glycerine, or, after drying, in Canada balsam. One may, however, use a contrast color,—such as methyl blue for the ground work. It is only necessary to float the cover glass, with the film downward, upon a one to two per centum watery solution of the methyl blue for five minutes. The excess of blue color is washed off with water, the cover glass dried and mounted. The bacilli of tuberculosis will be seen stained red and other elements will be blue.

For tissue containing the bacilli it is necessary to immerse the sections for from twelve to twenty-four hours in the fuchsin solution. They are then decolorized by immersion in the acid solution, the alcohol, etc., until only a faint redness remains. The sections are then dehydrated in alcohol and cleared up in oil of cloves. When mounted the bacilli are seen red, the tissue decolorized. The methyl blue may be used as a contrast color, also, for sections.

To easily detect the bacilli so prepared, one should have a microscope magnifying at least 450 diameters; however, the bacilli may be seen with a less powerful glass. An ordinary stand and substage will do for cover glass preparations, but an Abbé substage condenser is a decided aid to the discovery of the bacilli in cover glass preparations, and it is absolutely necessary in examining sections.

The discovery of the bacillus tuberculosis in the excretions, secretions or exudates examined is positively diagnostic of a tubercular disease. When it cannot be detected its absence is not of much diagnostic value, for it may be present in such small numbers as to render its detection difficult or impossible. When it is not found readily, repeated examinations of material collected on different days must be made to make its absence of any importance as a negative sign.

The bacillus is most easily detected in the supta of tuberculosis pulmonum. It is most difficult to detect it in the blood, even in cases of acute general tuberculosis.

It is not usually difficult, as a rule, to detect it in the exudates into serous cavities; as in tubercular pleuritis, tubercular peritonitis and tubercular synovitis. I have found the bacillus in the contents of a distended fallopian tube.

In tubercular disease of bone it is usually present in the cheesy infiltrate, but it is difficult to find it in the pus from sinuses in tubercular disease of bone.

It is difficult to detect the bacillus in the urine because of the bulk and the decomposing effect it has on the bacillus. *Then the preputial and labial smegma bacillus* gives the same color reaction as the tubercle bacillus, and its form is so nearly like the bacillus of tuberculosis that it cannot be differentiated from it, with the microscope. The presence, therefore, of a bacillus in secretions from the genitals, giving the color reaction and presenting the form of the bacillus tuberculosis, is not here, as it is elsewhere, a positive sign of tuberculosis.

In tubercular disease of the skin and mucous membranes the secretion therefrom sometimes contains the bacillus. Sections made from tissue taken from tubercular ulcers usually yield the bacillus.

The bacillus of leprosy is nearly like the tubercle bacillus in form, and it gives the same color reaction. The clinical course of leprosy is so distinct, and the disease so rare in this climate that it is not difficult to exclude it when considering a tubercular disease.

235 State Street.

